# Spironolactone is effective in treating hypokalemia among peritoneal dialysis patients

**DOI:** 10.1371/journal.pone.0187269

**Published:** 2017-11-10

**Authors:** Amit Langote, Swapnil Hiremath, Marcel Ruzicka, Brendan B. McCormick

**Affiliations:** 1 Department of Medicine, Division of Nephrology, Apollo Hospitals Enterprise Limited, Mumbai, India; 2 Department of Medicine, Division of Nephrology, Ottawa Hospital, Ottawa, Ontario, Canada; The University of Tokyo, JAPAN

## Abstract

**Background:**

Hypokalemia is common in peritoneal dialysis (PD) patients and is associated with increased cardiovascular and all-cause mortality. The management approach for such patients routinely includes spironolactone at our centre. We undertook this study to assess the efficacy of spironolactone for the treatment of hypokalemia in PD patients.

**Methods:**

Retrospective chart review of PD patients at a single centre. Serum potassium was compared prior to initiation of spironolactone and two months afterwards. Indication for spironolactone and changes in blood pressure (BP), weight, and serum creatinine were also recorded.

**Results:**

The chart review identified 53 patients who fit our selection criteria. The mean age was 64 +/- 15 years and the majority was treated with continuous cyclic peritoneal dialysis. Serum potassium rose from 3.7 +/- 0.5 to 4.2 +/- 0.5 mmol/L (P<0.0001) after 2 months with a mean dose of spironolactone of 28.5+/-15.2 mg (median dose 25 mg). A significant reduction in systolic BP was observed from 150+/- 18 to 137 +/-24 (P = 0.002); a non- significant reduction in diastolic BP was also observed. The rise in potassium was constant in the range of 0.4 to 0.5 mmol/L regardless of whether spironolactone was initiated for hypokalemia, diuresis, or as an antihypertensive. There was no change in serum creatinine or body weight two months after introduction of spironolactone.

**Conclusions:**

Spironolactone is safe and effective in treating hypokalemia in PD patients. It is also an effective antihypertensive agent and merits further study in the PD population.

## Introduction

Hypokalemia is a common occurrence in peritoneal dialysis (PD) patients and is reported to affect 10–36% of continuous ambulatory peritoneal dialysis (CAPD) patients [1–3]. Its prevalence may be even higher in continuous cyclic peritoneal dialysis (CCPD) patients due to the higher volume of dialysate and higher clearance of potassium with CCPD [4]. Hypokalemia is associated with increased cardiovascular and all-cause mortality, increased risk of peritonitis with enteric organisms, and malnutrition in PD patients[5–8] The exact cause of hypokalemia in these patients is not known and postulated to be due to a combination of decreased potassium intake due to dietary restriction, increased colonic potassium secretion in chronic kidney disease (CKD) patients and higher plasma insulin concentrations in response to peritoneal glucose loading which results in an intracellular potassium shift[9–10]

Hypokalemia is usually treated with oral potassium supplements but this is often limited due to side effects including gastritis and increased pill burden, which often leads to non-compliance with these medications. In our PD centre, we have long used the aldosterone antagonist, spironolactone, both for treatment of hypokalemia, and as a potassium sparing diuretic in our PD patients. This practice has been supported initially by our own experiences with spironolactone and more recently by literature supporting the safety of spironolactone in PD patients [11,12]. We undertook this retrospective review as a quality assurance project to determine the efficacy of spironolactone in increasing serum potassium values among PD patients.

## Material and methods

### Study population

The home dialysis program at the Ottawa Hospital serves a catchment area of approximately 1 million and follows approximately 170 home PD patients, the majority of whom are treated with CCPD. Patients are seen in clinic every two months by a multidisciplinary team that includes a home dialysis nurse, a nephrologist, a dietitian, and a social worker. Hypokalemia is managed in a stepwise fashion, initially with dietary advice, followed by spironolactone, and lastly potassium supplements which are used only if the potassium remains low, or if spironolactone is contraindicated or not tolerated. In addition, diuretics are used among those with residual urine output to maintain euvolemia and to avoid the use of hypertonic PD exchanges. Loop diuretics are prescribed in doses up to 320 mg daily of furosemide or equivalent. The choice of a second diuretic takes into account the serum potassium level on loop diuretics. When the potassium is less than 4 mmol/L spironolactone is added, and when greater than 4 mmol/L a thiazide-like diuretic is added. We treat hypertension by optimizing volume status and then starting renin angiotensin system (RAS) blockers, calcium channel blockers and beta blockers, in that sequence, unless there is any specific indication for any drug class. We consider spironolactone to be a fourth line antihypertensive.

### Chart review

Nephrocare (Fresenius, Bad Homberg Germany) is used as a data repository and electronic chart for the entire Ottawa Hospital dialysis program and includes hemodialysis and PD patients. All medication changes are inputted by the nurse at the time the medication is ordered by the nephrologist.

We searched Nephrocare using the key search word “spironolactone" between January 2001 and July 2013. We excluded patients who had not undergone PD, those who were taking spironolactone prior to starting PD, those who did not fill the prescription, those who transferred to hemodialysis (HD) in less than 2 months of PD start, those with missing laboratory data, and those who had concomitant changes to doses of oral potassium supplements, diuretics, RAS blockers, or to their PD prescription during the study period.

Once identified, baseline data including age, gender, race, PD vintage, presence of hypertension, diabetes, indications for spironolactone use, dose of spironolactone, and dose of furosemide were collected. Any medications affecting potassium balance were noted. Serum potassium, serum creatinine, blood pressure and weight, before and after 2 months of spironolactone start, were analyzed. Glomerular filtration rate (GFR) was calculated as mean of urea and creatinine clearance based on 24 hour urine collection.

### Statistical analysis

The primary outcome was change in potassium 2 months after initiation of spironolactone. Secondary outcomes included change in potassium based on indication for use, and change in body weight, creatinine, and blood pressure. Results are expressed as mean ± standard deviation or as median and inter-quartile range (IQR) unless otherwise specified. Pre and post-spironolactone data were compared using paired Student’s t test. A two-tailed p value of less than 0.05 was considered statistically significant. All analysis was carried out using Microsoft Excel 2010(Microsoft Inc. Redmond WA) and JMP (version 8.0.1, SASA Inc, Cary, NC).

## Results

**Fig 1:**
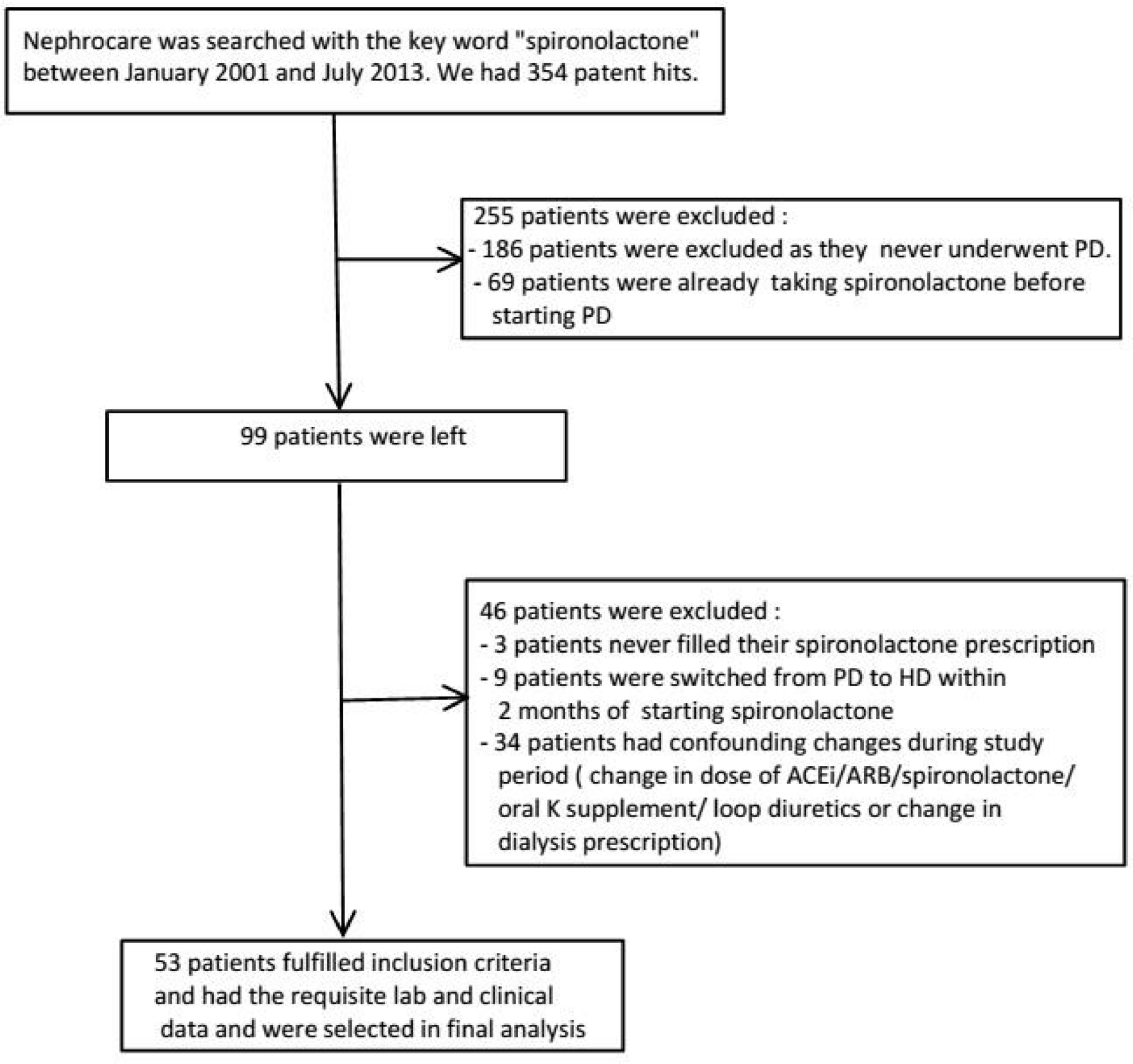
Patient flow.

Of the 354 patients identified by our initial search, 99 patients were started on spironolactone while on PD (see Fig 1). Thirty four patients were excluded as they had changes in dialysis prescription dose, dose of spironolactone, angiotensin converting enzyme inhibitor (ACEi), angiotensin receptor blocker (ARB), oral potassium supplement or loop diuretics during the 2 month study period. In addition, three patients never filled their spironolactone prescription and nine patients underwent switch of modality to HD within the study period and were excluded. Thus, 53 patients were included in the final analysis.

The baseline demographics of the 53 study patients are shown in Table 1. It was a predominantly white population with average age of 64 +/- 15 years. Most patients had a history of diabetes (58%) and hypertension (96%). Most patients were on cycler therapy and their median PD vintage was 25 months (IQR 14–38 months). They were on a high dose of furosemide (mean 149.4 +/- 103.82 mg) and their median dose of spironolactone was 25 mg.

**Table 1:** Demographics.

	N = 53
Age (years)	64.4 +/- 14.6
Male gender	30 (57%)
White Race	39 (74%)
Diabetes	31 (58%)
History hypertension	51 (96%)
Mean glomerular filtration rate (mL/min)	4.8 +/- 3.2
Mean urine volume (mL)	871 +/-697
Mean total Kt/V urea	2.27 +/- 0.51
CCPD	37 (70%)
Mean PD vintage (months)	29.5 +/- 18.7
Use of ACEi/ARB	28 (53%)
Use of potassium supplements	15 (28%)
Median dose of potassium chloride (mmol/day)	0 (IQR 0–20)
Median dose of spironolactone (mg)	25 (IQR 25–25)
Mean dose of furosemide (mg)	149.4 +/- 103.8

The majority of patients (33/53, 62%) treated with spironolactone had low potassium (<3.8 mmol/L). When examined by indication, the most common indication for use of spironolactone was as both hypokalemia and diuresis (n = 23, 43%), followed by purely as a diuretic (n = 9, 17%). The other indications for use were diuretic with antihypertensive effect (n = 7, 13%), treatment of isolated hypokalemia (n = 6, 11%), as antihypertensive (n = 4, 8%), and for treatment of hypokalemic hypertensive patients (n = 4, 8%).

### Potassium and BP changes in the entire population

Spironolactone caused a highly significant increase in the potassium level of 0.5 +/-0.5 mmol/L (p <0.0001) in the entire study population (Table 2). The change in potassium was similar irrespective of the indication for which it was used. The highest potassium recorded in the population was 5.2 mmol/L. None of the 9 patients who were excluded due to modality switch were documented to have developed hyperkalemia.

**Table 2:** Pre and post spironolactone changes by indication.

	entire population	(n = 53)		for hypokalemia	(n = 33)		as a diuretic	(n = 39)	
	Pre	Post	P value	Pre	Post	P value	Pre	Post	P value
K (mmol/l)	3.7 +/- 0.4	4.2+/-0.5	<0.0001	3.5+/-0.4	4.0+/-0.4	<0.0001	3.8+/-0.42	4.2+/-0.49	<0.00001
Creat (mmol/l)	698. +/-300	699.+/-279	0.99	622+/-204	632+/-169	0.55	629+/-186	635+/-231	0.84
SBP (mm Hg)	150+/- 18	137 +/-24	0.002	151+/-18	144+/-22	0.078	150+/-19	139+/-26	0.0052
DBP (mm Hg)	83.6+/-14.41	77.9+/-13.51	0.08	82+/-14	79+/-13	0.15	83+/-14	77+/-12.2	0.011
Weight (kg)	85.4+/-17.8	85.4+/-17.6	0.98	84.6+/-17.9	84.9+/-17.5	0.57	87.3+/-18.0	87.2+/-17.7	0.86

Spironolactone also significantly reduced systolic blood pressure (p = 0.002) with a non- significant reduction in diastolic pressure (p = 0.08). There was no change in creatinine or body weight in these patients suggesting that there was no change in residual kidney function, and that spironolactone did not achieve a significant diuretic effect. Table 3 shows concomitant use of medication by indication for spironolactone.

**Table 3:** Medication use by indication.

	Entire group	For hypokalemia	As a diuretic
Use of potassium supplements	15/53 (28%)	9/33 (27%)	11/39 (28%)
Median dose potassium chloride (mmol/day)	20 (IQR 0–20)	0 (IQR 0–20)	0 (IQR 0–20)
Use of furosemide	44/53 (83%)	26/33 (79%)	37/39 (95%)
Mean dose furosemide (mg/day)	149 +/- 103	137 +/- 107	178 +/-93
Median dose spironolactone (mg/day)	25 (IQR 25–25)	25 (IQR 25–25)	25 (IQR 25–25)
Use of ACEi/ARB	28/53 (52%)	14/33 (42%)	18/39 (46%)

### Use in hypokalemic patients

When used for any hypokalemia treatment, spironolactone use was associated with a significant increase in potassium by 0.5 +/-0.4 mmol/L (p <0.0001) but as shown in Table 2, the decrease in blood pressure was not statistically significant and the weight did not change. The median dose of spironolactone used for treatment of any hypokalemia was 25 mg.

#### Use as an antihypertensive

When used as an antihypertensive in 15 patients, at a median dose of 25 mg, there was an associated significant decline in systolic blood pressure from 151 +/- 17 to 124 +/- 23 mm Hg (p = 0.02) with a near significant decrease in diastolic pressure from 88 +/-16 to 78+/- 17 mm Hg (p = 0.059) and significant increase in serum the potassium 4.0 +/- 0.4 to 4.5 +/- 0.4 (p<0.001).

#### Use as a diuretic

When used as a diuretic (Table 2), at a median dose of 25 mg, there was an associated rise in potassium (p<0.00001) with significant decline in systolic (p = 0.005) and diastolic blood pressure (p = 0.011). However, there was no change in weight of the patients implying that there was no net diuretic effect.

## Discussion

Our retrospective chart review shows that in our practice, spironolactone is effective in raising serum potassium in PD patients regardless of the indication for use. Among patients treated for low serum potassium, it was effective in raising potassium to a normal range. It is also an effective antihypertensive agent when used in this population, though, as a diuretic it appears to have low potency and be essentially ineffective in reducing body weight at the doses used in our practice.

These findings are important as this is the first real world study to show that the strategy of using spironolactone to treat PD patients with hypokalemia and/or hypertension can be quite effective from both a potassium and blood pressure perspective. To date, results from randomized controlled trials of spironolactone in PD have been somewhat conflicting with respect to the ability of spironolactone to effect serum potassium levels[11–14]

Four randomized trials have been conducted using spironolactone in the PD population[11–14] Two of them, one from Hong Kong (pre and post potassium were 3.30 ± 0.26 vs. 3.46 ± 0.38 mmol/L, p = 0.28), and one from Thailand (pre and post potassium were 3.90±0.59 vs. 4.23±0.64 mmol/L, P = 0.077) included hypokalemic patients and were negative studies ([13,14]. The 2007 Hong Kong study did not find any difference in serum potassium and blood pressure after treating 12 hypokalemic continuous ambulatory peritoneal dialysis (CAPD) patients with 25 mg spironolactone for 6 months[13]. Similarly the Thailand study did not find any effect of 25 mg spironolactone, given for 4 weeks, on serum potassium and urinary excretion of potassium in 20 hypokalemic CAPD patients [14]. Both these studies were very small and included patients on CAPD only, while our patient population was predominantly CCPD. A third study by Taheri et al. randomized 18 normokalemic CAPD patients with congestive heart failure to either placebo or spironolactone 25 mg alternate day[12] The serum potassium rose modestly in both groups but did not achieve statistical significance. Unlike our study, the above studies did not include a formal dietary counselling to increase dietary potassium intake and adopt a high potassium diet. It is therefore possible that spironolactone is only effective in raising potassium in conjunction with an increased potassium intake. The fact that all of our hypokalemic patients had previously received dietary counselling prior to starting spironolactone argues that the increase in serum potassium was not due to the dietary counselling alone but perhaps the combination of the two.

The largest randomized controlled trial of spironolactone in PD patients was reported by Ito et al and it did report significantly higher levels of potassium at 6 and 12 months among 78 PD patients treated with spironolactone compared with control[11]. The absolute difference of serum potassium between the two groups was in the range of 0.4 mmol/L, similar to our findings. In this trial, spironolactone also prevented cardiac hypertrophy and decreases in left ventricular ejection fraction in patients undergoing PD, without significant adverse effects. A recent observational study by Fulop et al. has demonstrated significantly reduced use of oral potassium supplementation among patients treated with potassium sparing diuretics, mostly spironolactone[15].

Spironolactone also appears to safely raise serum potassium in HD patients, even those with anuria[16]. Matsumoto et al., in a study of 61 HD patients, reported an average increase in pre-dialysis potassium of 0.3 mmol/L, after daily use of 25 mg spironolactone for 2 weeks[17]. A similar increase in potassium was reported by Gross et al after use of high dose spironolactone, 50 mg twice daily, for a period of 2 weeks in chronic HD patients [16]. Recently, a longer-term study in HD patients used 25 mg spironolactone daily for 3 years and showed a significant risk reduction in cardiovascular and cerebrovascular morbidity and mortality [18]. However, there was no change in potassium levels or blood pressure in the spironolactone group. Lin et al. reported a similar reduction in cardiovascular mortality in a randomized controlled trial and reported a non-significant rise in potassium in the spironolactone group. [19]. A recent meta-analysis of randomized controlled trials of mineralocorticoid antagonists in dialysis patients confirmed an apparent reduction in cardiovascular and all-cause mortality and an increase in risk of hyperkalemia. The authors did however have methodological concerns and felt that a large randomized controlled trial was needed to further clarify the risks and benefits of mineralocorticoid antagonists in dialysis patients. [20]

In our study, spironolactone also proved to be an effective antihypertensive agent. There was a significant decline in systolic blood pressure of 13 mm Hg while the diastolic pressure reduction (mean 6 mm Hg) was close to significant. Three of the four randomized trials of spironolactone in PD patients report no significant change in BP, while the fourth does not comment on any change in blood pressure. The baseline blood pressure in these studies was lower than ours. Again, however, our findings of a hypotensive effect of spironolactone more closely parallels what has been reported in the HD literature. Gross et al. demonstrated a reduction in systolic blood pressure by spironolactone (from 143 ± 10 to 130 ± 22 mm Hg, P < 0.05) but not in diastolic blood pressure [16]. Similarly, Shavit et al. also showed reduction in systolic blood pressure (166 ± 14 to 153 ± 10 mm Hg, P < 0.05) in HD patients with the mineralocorticoid receptor antagonist eplerenone[21].

The exact cause of hypokalemia in PD patients is not known and postulated to be due to decreased potassium intake due to dietary restriction, increased colonic potassium secretion in CKD and ESRD patients [9,10]Colonic potassium secretion is enhanced through the increase in apical potassium permeability of the large intestinal epithelium in anuric ESRD patients[9]. Metabolic potassium balance studies among normokalemic CAPD patients have shown that 17–32% of dietary potassium is lost in the stool. Overall 24 hour potassium balance among these patients was slightly positive. [22] Tziviskou et al studied dietary potassium intake and urinary and peritoneal losses of potassium among four severely hypokalemic CAPD patients and found an apparent positive balance of 20 mmol/day. [23] They did not measure gastrointestinal losses of potassium but stool losses of 17 to 20 mmol/day were described in the study by Blumenkrantz et al. so it is difficult to conclude if hypokalemic PD patients are actually in negative potassium balance.

Chronic hypokalemia in PD patients if often attributed to an increased intracellular shift of potassium due, perhaps due to hyperinsulinemia. Muscle biopsy studies have shown that the muscle concentration of potassium is elevated in PD patients [24]. It follows that if hypokalemic PD patients are indeed in even potassium balance, as suggested by Tziviskou et al [23],and intracellular potassium concentration is increased then it is expected that there would be a concomitant decrease in extracellular potassium.

The demonstrated increase in serum potassium with spironolactone could be due to either reduced potassium losses, or altered transcellular potassium shift. Mineralocorticoid receptors in the colonic epithelium cells are thought to play an important role in this potassium elimination. Spironolactone appears to act by inhibiting these mineralocorticoid receptors and thereby preventing colonic potassium losses and causing rise in serum potassium levels. [25] In addition, many of our patients had significant urinary volumes and a decrease in urinary potassium losses may also be important. On the other hand, skeletal muscle cells contain aldosterone receptors and aldosterone blockade has also been shown to markedly affect the ability of CKD patients to shift potassium into cells when angiotensin converting enzyme inhibitors are used concurrently and is an important reason why patients with CKD develop hyperkalemia with dual renin-angiotensin-aldosterone axis blockade. [26,27] Thus, we can only speculate whether the observed increase in potassium in our patients was due to decreased bowel and urinary excretion or diminished intracellular shift.

Our study has a number of limitations. It is a retrospective chart review and a large number of patients had to be excluded from analysis due to confounding changes in medications and prescriptions. We report only short term outcomes at 2 months. This time point was chosen to minimize other confounding changes in medications and PD prescription which would have occurred if a later time point with longer follow up time accrued was chosen. Our patients also received dietary counselling to increase potassium intake, usually several weeks prior to the initiation of spironolactone. Thus, our reported effect of spironolactone on serum potassium may be best classified as the combined effect of spironolactone and dietary counselling to increase potassium intake. The observed blood pressure effect was much greater than expected based on the existing PD literature, though similar to what has been reported in oliguric HD patients[16]. Future studies of spironolactone in PD patients should use the gold standard of 24 hour ABPMs to better assess for this BP effect as it may be missed by relying simply on clinic blood pressures. A further limitation is that medication compliance was confirmed only by interviewing the patient and not by medication count or filling history report from the pharmacy.

Despite the limitations, our study suggests that spironolactone may be a useful agent to correct hypokalemia and treat hypertension in PD patients. To better understand the therapeutic potential of spironolactone in PD, we recommend that larger randomized controlled trials assessing both treatment of hypokalemia and treatment of hypertension be performed in this population.

## References

[1] Spital A, Sterns RH. Potassium supplementation via the dialysate in continuous ambulatory peritoneal dialysis. American journal of kidney diseases: the official journal of the National Kidney Foundation. 1985;6:173–6. 3898826 10.1016/s0272-6386(85)80022-6

[2] Oreopoulos DG, Khanna R, Williams P, Vas SI. Continuous ambulatory peritoneal dialysis—1981. Nephron. 1982;30:293–303. 7110460 10.1159/000182504

[3] Khan AN, Bernardini J, Johnston JR, Piraino B. Hypokalemia in peritoneal dialysis patients. Perit Dial Int. 1996;16:652 8981546

[4] Gao H, Lew SQ, Bosch JP. Biochemical parameters, nutritional status and efficiency of dialysis in CAPD and CCPD patients. Am J Nephrol. 1999;19:7–12. doi: 10.1159/000013418 10085443 10.1159/000013418

[5] Torlen K, Kalantar-Zadeh K, Molnar MZ, Vashistha T, Mehrotra R. Serum potassium and cause-specific mortality in a large peritoneal dialysis cohort. Clinical journal of the American Society of Nephrology: CJASN. 2012;7:1272–84. doi: 10.2215/CJN.00960112 22626960 10.2215/CJN.00960112 PMC3408121

[6] Dolson GM. Do potassium deficient diets and K+ removal by dialysis contribute to the cardiovascular morbidity and mortality of patients with end stage renal disease? Int J Artif Organs. 1997;20:134–5. 9151147

[7] Chuang YW, Shu KH, Yu TM, Cheng CH, Chen CH. Hypokalaemia: an independent risk factor of Enterobacteriaceae peritonitis in CAPD patients. Nephrol Dial Transplant. 2009;24:1603–8. doi: 10.1093/ndt/gfn709 19103738 10.1093/ndt/gfn709

[8] Szeto CC, Chow KM, Kwan BC, Leung CB, Chung KY, Law MC, et al Hypokalemia in Chinese peritoneal dialysis patients: prevalence and prognostic implication. American journal of kidney diseases: the official journal of the National Kidney Foundation. 2005;46:128–35. 10.1053/j.ajkd.2005.03.015 15983966

[9] Mathialahan T, Maclennan KA, Sandle LN, Verbeke C, Sandle GI. Enhanced large intestinal potassium permeability in end-stage renal disease. J Pathol. 2005;206:46–51. doi: 10.1002/path.1750 15772943 10.1002/path.1750

[10] Sandle GI, Gaiger E, Tapster S, Goodship TH. Evidence for large intestinal control of potassium homoeostasis in uraemic patients undergoing long-term dialysis. Clin Sci (Lond). 1987;73:247–52. 3652631 10.1042/cs0730247

[11] Ito Y, Mizuno M, Suzuki Y, Tamai H, Hiramatsu T, Ohashi H, et al Long-term effects of spironolactone in peritoneal dialysis patients. J Am Soc Nephrol. 2014;25:1094–102. doi: 10.1681/ASN.2013030273 24335969 10.1681/ASN.2013030273 PMC4005296

[12] Taheri S, Mortazavi M, Pourmoghadas A, Seyrafian S, Alipour Z, Karimi S. A prospective double-blind randomized placebo-controlled clinical trial to evaluate the safety and efficacy of spironolactone in patients with advanced congestive heart failure on continuous ambulatory peritoneal dialysis. Saudi J Kidney Dis Transpl. 2012;23:507–12. 22569436

[13] Kwok JS-S, Chow K-M, Kwan BC-H, Li PK-T, Szeto C-C. Spironolactone is not Effective for the Treatment of Hypokalemia in Peritoneal Dialysis Patients. Hong Kong Journal of Nephrology.9:36–40.

[14] Yongsiri S, Thammakumpee J, Prongnamchai S, Tengpraettanakorn P, Chueansuwan R, Tangjaturonrasme S, et al Randomized, Double-Blind, Placebo-Controlled Trial of Spironolactone for Hypokalemia in Continuous Ambulatory Peritoneal Dialysis Patients. Therapeutic Apheresis and Dialysis. 2015;19:81–6. doi: 10.1111/1744-9987.12219 25196890 10.1111/1744-9987.12219

[15] Fülöp T, Zsom L, Rodríguez B, Afshan S, Davidson JV, Szarvas T, Dixit MP, Tapolyai MB, Rosivall L. Clinical utility of potassium-sparing diuretics to maintain normal serum potassium in peritoneal dialysis patients. Peritoneal Dialysis International. 2017;37:63–9. doi: 10.3747/pdi.2016.00022 27282853 10.3747/pdi.2016.00022

[16] Gross E, Rothstein M, Dombek S, Juknis HI. Effect of spironolactone on blood pressure and the renin-angiotensin-aldosterone system in oligo-anuric hemodialysis patients. American journal of kidney diseases. 2005;46:94–101. 15983962 10.1053/j.ajkd.2005.03.005

[17] Matsumoto Y, Mori Y, Kageyama S, Arihara K, Sugiyama T, Ohmura H, et al Spironolactone reduces cardiovascular and cerebrovascular morbidity and mortality in hemodialysis patients. J Am Coll Cardiol. 2014;63:528–36. doi: 10.1016/j.jacc.2013.09.056 24184249 10.1016/j.jacc.2013.09.056

[18] Matsumoto Y, Kageyama S, Yakushigawa T, Arihara K, Sugiyama T, Mori Y, et al Long-term low-dose spironolactone therapy is safe in oligoanuric hemodialysis patients. Cardiology. 2009;114:32–8. doi: 10.1159/000210553 19342857 10.1159/000210553

[19] Lin C, Zhang Q, Zhang H, Lin A. Long Term Effects of Low Dose Spironolactone on Chronic Dialysis Patients: A Randomized Placebo Controlled Study. The Journal of Clinical Hypertension. 2016;18:121–8. doi: 10.1111/jch.12628 26224543 10.1111/jch.12628 PMC8031645

[20] Quach K, Lvtvyn L, Baigent C, Bueti J, Garg AX, Hawley C, Haynes R, Manns B, Perkovic V, Rabbat CG, Wald R. The safety and efficacy of mineralocorticoid receptor antagonists in patients who require dialysis: a systematic review and meta-analysis. American Journal of Kidney Diseases. 2016;68:591–8. doi: 10.1053/j.ajkd.2016.04.011 27265777 10.1053/j.ajkd.2016.04.011

[21] Shavit L, Neykin D, Lifschitz M, Slotki I. Effect of eplerenone on blood pressure and the renin-angiotensin-aldosterone system in oligo-anuric chronic hemodialysis patients—a pilot study. Clin Nephrol. 2011;76:388–95. 22000559 10.5414/cn106973

[22] Blumenkrantz MJ, Kopple JD, Moran JK, Coburn JW. Metabolic balance studies and dietary protein requirements in patients undergoing continuous ambulatory peritoneal dialysis. Kidney international. 1982;21:849–61. 7132054 10.1038/ki.1982.109

[23] Tziviskou E, Musso C, Bellizzi V, Khandelwal M, Wang T, Savaj S, Oreopoulos DG. Prevalence and pathogenesis of hypokalemia in patients on chronic peritoneal dialysis: one center's experience and review of the literature. International urology and nephrology. 2003;35:429–34. 15160552 10.1023/b:urol.0000022867.93739.03

[24] Bergström J, Alvestrand A, Fürst P, Hultman E, Widstam-Attorps U. Muscle intracellular electrolytes in patients with chronic uremia. Kidney international. Supplement. 1983;16:S153–60. 6588246

[25] Guerrant RL, Chen LC, Rohde JE. Effect of spironolactone on stool electrolyte losses during human cholera. Gut. 1972;13:197–200. 5024724 10.1136/gut.13.3.197 PMC1412119

[26] Unwin RJ, Luft FC, Shirley DG. Pathophysiology and management of hypokalemia: a clinical perspective. Nature Reviews Nephrology. 2011;7:75–84. doi: 10.1038/nrneph.2010.175 21278718 10.1038/nrneph.2010.175

[27] Preston RA, Afshartous D, Garg D, Medrano S, Alonso AB, Rodriguez R. Mechanisms of impaired potassium handling with dual renin-angiotensin-aldosterone blockade in chronic kidney disease. Hypertension. 2009;53:754–60. doi: 10.1161/HYPERTENSIONAHA.108.125252 19307466 10.1161/HYPERTENSIONAHA.108.125252

